# Imagens Complementam Palavras: Ausência de Progressão de Lesão Miocárdica e Coronariana em Crianças em Idade Escolar submetidos à Ablação por Cateter de Radiofrequência

**DOI:** 10.36660/abc.20230850

**Published:** 2024-03-06

**Authors:** Jorge Elias, Márcio Augusto Silva

**Affiliations:** 1 Serviço de Eletrofisiologia Vitória Apart Hospital Serra ES Brasil Vitória Apart Hospital - Serviço de Eletrofisiologia, Serra, ES - Brasil; 2 Universidade Federal do Espírito Santo Serviço de Eletrofisiologia Vitória ES Brasil Universidade Federal do Espírito Santo (UFES) - Serviço de Eletrofisiologia, Vitória, ES - Brasil

**Keywords:** Ablação por Cateter, Lesão Miocárdica, Pediátrico, Segurança

Nem mesmo os maiores entusiastas entre os cardiologistas poderiam prever, no final da década de 1980, o impacto que a ablação por cateter teria no tratamento das arritmias cardíacas. Desde o seu advento acumularam-se evidências e experiência clínica que permitem afirmar a sua eficácia no tratamento das mais variadas formas de taquiarritmias cardíacas em indivíduos de todas as faixas etárias.^1 - 3^

Particularmente na população pediátrica, essas perspectivas mudaram gradualmente do uso de ablação por cateter apenas em casos de arritmia refratária a medicamentos para um tratamento de primeira linha e preferência do paciente/familiares.^2 , 4 - 7^ No entanto, essa generalização levanta um questionamento referente ao uso dessa técnica na prática clínica na ausência de dados em animais ou humanos que demonstrem suficientemente sua segurança e eficácia em órgãos imaturos.^8 , 9^

O manejo das arritmias pediátricas é potencialmente desafiador, embora, em crianças sem doença cardíaca congênita, os mecanismos das arritmias sejam os mesmos que nos adultos.^6^ Esta observação é particularmente relevante em pacientes com menor peso corporal e naqueles com doença cardíaca congênita.^2 , 6^

Desde o advento da ablação por cateter, os autores demonstraram preocupação sobre o potencial de lesão das estruturas cardíacas, mediada por trauma mecânico do cateter ou por energia ablativa.^2 , 10^

Pacientes pediátricos podem ter maior probabilidade de apresentar lesões estruturais devido às paredes miocárdicas mais finas, válvulas mais delicadas e miocárdio imaturo.^10^

Apesar disso, mesmo em adultos, o objetivo final de analisar as complicações da ablação por cateter restringe-se à fase aguda, com as complicações usuais como bloqueio atrioventricular, perfuração cardíaca, tromboembolismo, derrame pericárdico, dano valvar e, raramente, lesão arterial coronariana.^11^ Com relação ao acompanhamento em longo prazo, os estudos geralmente se restringem à recorrência da taquiarritmia ou à progressão para bloqueio atrioventricular.^1 , 4 , 12 - 14^

Dentre as complicações tardias estudadas em pacientes jovens, podemos destacar a influência psicológica das taquiarritmias,^15 , 16^ o efeito da exposição à radiação e o potencial impacto na ocorrência de futuras neoplasias^17^ e na potencial progressão da lesão miocárdica causada pela radiofrequência.^18^

Os dados relativos à formação de lesões induzidas pela descarga de corrente de radiofrequência no miocárdio imaturo são esparsos e controversos.

A ressonância magnética (RM) consolidou-se como um parceiro importante na definição do manejo e no monitoramento da evolução das doenças cardíacas e, mais recentemente, os avanços na tecnologia de imagem e no pós-processamento estão facilitando o uso de imagens avançadas antes, durante e após a ablação em pacientes com arritmias atriais e ventriculares. Além disso, em pacientes com arritmia recorrente pós-ablação, o realce tardio com gadolínio pode potencialmente identificar alvos para repetição da ablação.^19 - 22^

Pensando nisso, Melo et al.,^18^ de forma inédita, realizando RM e angiografia coronariana após um tempo mediano de 6,7 anos desde a ablação, buscaram avaliar achados tardios secundários à radiofrequência em uma coorte de pacientes que realizaram tratamento das formas mais comuns de taquicardia paroxística supraventricular na infância.^18^ Os autores demonstraram que a RM, realizada com cateteres não irrigados na infância e adolescência, foi associada à presença de pequenas quantidades de fibrose miocárdica sem qualquer impacto na evolução clínica dos pacientes.^18^ Esses achados levaram os autores a considerar que a ablação por radiofrequência não aumenta as chances de arritmias ou disfunção do ventrículo esquerdo durante o acompanhamento e desenvolvimento dessas crianças.^18^

De qualquer forma, como bem observado pelos autores, esse estudo^18^ foi realizado em crianças em idade escolar, o que pode minimizar o impacto da evolução da fibrose quando comparado a uma população de pacientes mais jovens.

Saul et al., em estudo experimental, observaram que lesões por radiofrequência, realizadas em ovinos infantis e avaliadas de forma aguda e com intervalo de 8 semanas, parecem aumentar de tamanho com o passar do tempo. Os autores especularam que esses achados, que presumivelmente resultam de diferenças na resposta inflamatória ou no potencial de crescimento celular no miocárdio em desenvolvimento, podem ter implicações para o desempenho da ablação por radiofrequência em corações infantis.^23^

Em relação aos resultados diferentes dos estudos em animais comparados aos realizados em humanos, Melo et al. observaram que faltam dados comparando variações de idade e evolução entre diferentes espécies e sua potencial interferência fisiopatológica.^18^

Talvez por esta razão, um novo estudo experimental randomizado realizado em porcos jovens, que avaliou e comparou o crescimento das lesões durante o primeiro ano produzidas por ablação criotérmica e por cateter de radiofrequência em corações de bebês, reproduziu os resultados de Saul et al. e mostrou que em ambos os tipos de energia houve um grau de lesão transmural que progrediu de forma semelhante em nível atrial e ventricular, refutando a noção de que a crioablação deveria ser favorecida com base na expansão da lesão.^23 , 24^

Esse mesmo estudo demonstrou que os volumes das lesões do sulco AV não aumentam significativamente com nenhuma das modalidades de energia, o que torna a ablação por cateter mais viável, uma vez que a maioria dos casos em todas as idades é realizada para substratos relacionados ao sulco AV (71% em lactentes e 89% em crianças).^8^

Outra observação relevante foi a ausência de lesões coronarianas, sejam estenoses ou calcificações, documentadas por tomografia computadorizada.^18^

A lesão da artéria coronária após a ablação por cateter continua a ser uma preocupação, embora felizmente seja um evento raro. Essa complicação geralmente é aguda, causada por lesão térmica direta na artéria coronária que pode levar a espasmo arterial coronariano, trombose aguda, estenose e até oclusão crônica.^10^

Paul et al., também utilizando ovelhas jovens, observaram que na maioria dos animais estudados havia algum grau de comprometimento coronariano e levantaram a hipótese de um risco potencial de comprometimento da artéria coronária, ou seja, espessamento intimal que pode proporcionar um nicho para forças de cisalhamento e formação de placa ao longo dos anos de desenvolvimento.^25^

Da mesma forma que no presente estudo, esses achados não foram corroborados por Khairy et al., que não relataram efeitos de ablação em artérias coronárias próximas.^24^

Embora estejamos vivenciando um avanço tecnológico sem precedentes no campo da eletrofisiologia cardíaca, e também que os achados do estudo^18^ em questão não incluam pacientes de baixo peso, pacientes com arritmias complexas e pacientes com cardiopatias congênitas, devemos ter em mente que no mundo real, a maioria dos pacientes jovens submetidos à ablação por cateter de radiofrequência usando fluoroscopia, pelo menos no primeiro procedimento, são diagnosticados com taquicardia atrioventricular ou taquicardia de reentrada nodal na ausência de doença cardíaca subjacente. Ou seja, eles estão representados neste estudo.^18^

Novos estudos são necessários para avaliar o impacto de novas técnicas de ablação por cateter, que acrescentem, por um lado, novos cateteres (cateter irrigado com e sem sensor de contato) associados a novas técnicas de mapeamento (mapeamento 3D) e novos tipos de energia (crioablação e ablação por campo pulsado) sobre o risco de complicações agudas e o risco potencial de futura formação de cicatriz miocárdica.
