# Supplementary material for: Imagens Complementam Palavras: Ausência de Progressão de Lesão Miocárdica e Coronariana em Crianças em Idade Escolar submetidos à Ablação por Cateter de Radiofrequência
Source: Arq Bras Cardiol. 2024 Mar 6;121(1):e20230850. [Article in Portuguese] doi: 10.36660/abc.20230850 (PMC11081400; doi:10.36660/abc.20230850)
Supplement: Translation

# Translation: Images Complement Words: Absence of Progression of Myocardial and Coronary Artery Injury in School-Age Children Undergoing Radiofrequency Catheter Ablation

**Authors:** Jorge Elias, Márcio Augusto Silva

**Affiliations:** 1 Serra ES Brazil Vitória Apart Hospital - Serviço de Eletrofisiologia, Serra, ES - Brazil; 2 Vitória ES Brazil Universidade Federal do Espírito Santo (UFES) - Serviço de Eletrofisiologia, Vitória, ES - Brazil

**Keywords:** Catheter Ablation, ocardial Contusions, Pediatrics, Safety

Not even the biggest enthusiasts among cardiologists could predict, at the end of the 1980s, the impact that catheter ablation would have on the treatment of cardiac arrhythmias. Since its advent, evidence, and clinical experience have accumulated that allow to affirm its effectiveness in the treatment of the most varied forms of cardiac tachyarrhythmias in individuals of all age groups.^1 - 3^

Particularly in the pediatric population, these perspectives have gradually shifted from the use of catheter ablation only in cases of drug-refractory arrhythmia to a first-line treatment and patient/parent preference.^2 , 4 - 7^However, this generalization raises the question of whether it is appropriate to adopt a technique into clinical practice in the absence of animal or human data that sufficiently demonstrates both its safety and efficacy in immature organs.^8 , 9^

Management of pediatric arrhythmias is potentially challenging, although, in children without congenital heart disease, the mechanisms of arrhythmias are the same as in adults.^6^This observation is particularly relevant in patients of lower body weight and those with congenital heart disease.^2 , 6^

Since the advent of catheter ablation, authors have raised concerns about the potential for injury to cardiac structures, mediated either by mechanical catheter trauma or by ablative energy.^2 , 10^

Pediatric patients may be more likely to present with structural injuries due to their thinner myocardial walls, more delicate valves, and immature myocardium.^10^

Despite this, even in adults, the final objective of analyzing complications from catheter ablation is restricted to the acute phase, with the usual complications such as atrioventricular block, cardiac perforation, thromboembolism, pericardial effusion, valvular damage and, rarely, coronary artery injury.^11^With regard to long-term follow-up, studies are usually restricted to tachyarrhythmia recurrence or progression to atrioventricular block.^1 , 4 , 12 - 14^

Among the late complications studied in young patients, we can highlight the psychological influence of tachyarrhythmias,^15 , 16^the effect of exposure to radiation, and the potential impact on the occurrence of future neoplasia^17^and the potential progression of myocardial injury caused by radiofrequency.^18^

Data concerning lesion formation induced by radiofrequency current discharge in immature myocardium are sparse and controversial.

Magnetic resonance imaging (MRI) has established itself as an important partner in defining management and monitoring the evolution of heart disease and, more recently, advances in imaging technology and postprocessing are facilitating the use of advanced imaging before, during, and after ablation in patients with both atrial and ventricular arrhythmias. Furthermore, in patients with recurrent arrhythmia post-ablation, late gadolinium enhancement can potentially identify targets for repeat ablation.^19 - 22^

With this in mind, Melo et al.,^18^in an unprecedented way, performing MRI and coronary angiography after a median time from ablation of 6.7 years sought to evaluate late findings secondary to radiofrequency in a cohort of patients who underwent treatment of the most common forms of paroxysmal supraventricular tachycardia during childhood.^18^The authors demonstrated that MRI, performed with non-irrigated catheters in middle childhood and adolescence, was associated with the presence of small amounts of myocardial fibrosis without any impact on the patients’ clinical evolution.^18^These findings lead to consider that radiofrequency ablation does not increase the chances of arrhythmias or left ventricular dysfunction during follow-up and development of these children.^18^

In any case, as appropriately noted by the authors, this study^18^was carried out in school-age children, which may minimize the impact of fibrosis evolution when compared to a younger patient population.

Saul et al., in an experimental study, observed that radiofrequency lesions, performed on infant sheep and evaluated acutely and at an interval of 8 weeks, appear to increase in size over time. The authors speculated that these findings, which presumably result from differences in either the inflammatory response or the potential for cell growth in developing myocardium, may have implications for the performance of RF ablation in infant hearts.^23^

Regarding the different results of animal studies compared to those carried out in humans, Melo et al. observed that there is a lack of data comparing variations in age and evolution between different species and their potential pathophysiological interference.^18^

Perhaps for this reason, a new randomized experimental study carried out in young pigs now evaluates and compares the growth of lesions during the first year produced by cryothermal and radiofrequency catheter ablation in infant's hearts, reproducing the results of Saul et al. and showing that in both types of energy, a degree of transmural lesion that progressed similarly at the atrial and ventricular level, refuting the notion that cryoablation should be favored based on lesion expansion.^23 , 24^

This same study demonstrated that AV groove lesion volumes do not increase significantly with either energy modality, which makes catheter ablation more feasible since most cases at every age are performed for AV groove-related substrates (71% in infants and 89% in children).^8^

Another relevant observation was the absence of coronary artery lesions, either stenosis or calcification, documented by computed tomography.^18^

Coronary artery injury following catheter ablation remains a concern, although it is fortunately a rare event. This complication is usually acute, caused by direct thermal injury to the coronary artery that may lead to coronary artery spasm, acute thrombosis, stenosis, and even chronic occlusion.^10^

Paul et al., also using young sheep, observed that in most of the animals studied, there was some degree of coronary involvement and raised the hypothesis of a potential risk of the coronary artery being affected, that is, intimal thickening that may provide a nidus for shear forces and plaque formation over years of development.^25^

Similarly to the present study, these findings were not corroborated by Khairy et al., who did not report on ablation effects on nearby coronary arteries.^24^

Although we are experiencing an unprecedented technological advance in the field of cardiac electrophysiology, and also that the findings of the study^18^in question do not include low-weight patients, patients with complex arrhythmias, and patients with congenital heart disease, we must bear in mind that in the real world, most young patients undergo radiofrequency catheter ablation using fluoroscopy, at least in the first procedure, are diagnosed with atrioventricular tachycardia or nodal reentry tachycardia in the absence of underlying heart disease. In other words, they are represented in this study.^18^

New studies are needed to evaluate the impact of new catheter ablation techniques, which add, on the one hand, new catheters (irrigated catheter with and without contact sensor) associated with new mapping techniques (3D mapping) and new types of energy (cryoablation and pulsed-field ablation) on the risk of acute complications and the potential risk of future myocardial scar formation.
